# Keystone Veterinary Medical Association

**Published:** 1891-12

**Authors:** W. S. Kooker

**Affiliations:** Secretary


### Society Proceedings.

*Keystone Veterinary Medical Association.*—The regular meeting of the Keystone Veterinary Association, was held at the College of Physicians, Nov., 7, 1891. The President, W. Horace Hoskins, in the chair. Present, Drs. W. B. E. Miller, Chas. T. Goentner, Alex. Glass, W. H. Hoskins, J. B. Rayner, W. S. Kooker, Chas. M. Cullen, R. G. Webster and W. B. Werntz. Minutes of previous meeting were read and approved. The President filled vacancies in Board of Trustees by appointing Drs. Eves, Rayner, Miller and Goentner.

A recess was taken, and the Board, upon reconvening, reported adversly upon the application of Dr. MacFayden, for associate membership, and approved the resignation of Dr. M. W. Drake. On motion the report of the Board of Trustees, was accepted.

Dr. Glass, the essayist of the evening, read a paper on "Bench Show Distemper." Dogs gotten in the very pink of condition are sent from all parts of the country to the show. After or during the latter part of the show, they manifest a disinclination to feed, are drowsy, languid, the coat unthrifty in appearance, eyes watery, nose dry, rapid pulse, high temperature, with in some instances, cough. This constitues the general symptoms of Bench Show distemper in dogs; which we distinguish from the real or genuine distemper, by the symptoms in the majority of cases, being of a milder form. We have three different conditions resulting from Bench Show Distemper in dogs, which are of a serious nature, first, the Pneumonic, second, the Abdominal, third, the Nervous. In the first, or Pneumonic condition, we have all the symptoms seen in the same disease in other domestic animals. In the second, or Abdominal condition we have enteritic symptoms, with either a diarrhoea or dysentery, or the opposite, excessive constipation. This condition as well as the first condition requires very careful nursing, and rational treatment to bring about a good recovery. The last, or Nervous condition is the one in which the results of treatment are generally very unsatisfactory.

The cause of Bench Show Distemper, in a great measure, is brought about by change in the manner of both feeding and housing. Another source is no doubt, due to the fact, that these dogs are housed in one system of kennel, called the Spratts' Bench System. These wooden benches or cages are moved from one city to another throughout the whole country, and have only a mere superficial cleansing with water.

The percentage of cases of Bench Show Distemper would no doubt be much less if these kennels were thoroughly disinfected and more care given to the cleansing. Dr. R. G. Webster, then reported the following cases :

On the 12th of April, 1891, I was called to attend five cows of a man's herd, which he said were acting very strangely to him. I drove to his place at once, found the five cows in a stable, at first glance they appeared to have nothing the matter with them, as they were eating hay at the time; their coats were glossy, and in fair condition, but on a closer examination, found a very serious trouble. Accidently touching one upon the back, she almost fell to the ground, losing all power and control of herself, her legs were almost useless, she then showed a very wild look out of her eyes, as though in great distress; by taking my hand off she soon went to eating again, temperature and respiration were normal, pulse weak but not fast; could handle her anywhere over body, except spine, without her showing any distress. The history the owner gave was that the week before he had one old cow to get down, and could not rise again, he thinking she was paralized from old age killed her; thinking nothing more about it until four days later in going into the stable in the morning found the five as before mentioned. I found they were feeding upon malt-dust and clover-hay, and during the winter got it mixed with corn-meal, hut, his corn running out about a month before had been since then feeding the malt alone, was feeding one ton every ten days to twenty-seven head, soaking each feed for twelve hours in three hundred quarts of water. I looked at the hay and malt but could see nothing wrong with either, or anything about the barn or yard as everything was clean. Thinking the trouble might come from feeding malt too heavily I had it discontinued and gave tonics to the cows.

I looked at the rest of the herd in the lot before going away, they appeared to be all right. On the 13th, I did not see them, on the morning of the 14th, the owner was at my office by six o'clock reporting eight more effected in the same manner. I ordered him to send at once for State Veterinarian Dr. Bridge, which he did. I got to his place about ten in the morning, he had turned them out into the lot, I looked them over and found the whole twenty-seven more or less effected, two not being able to get out of the stable, one down not able to get upon her feet but was eating, was very hungry, gave her a bucket of water, drank it down without any trouble in the least, gave another bucket, drank it, wanted more, gave her some dry bran, she licked at it as any healthy cow would. Her temperature and respiration were normal, pulse weak; the other apparently the weaker of the two could get up and down, was hungry and thirsty as the first. If the cows were in the field they would be eating grass, then all at once would stop, stamp their feet, kick at belly and then start to run as if bees were stinging them, would elevate tail over back and run this way a few minutes, then stop and go to eating grass; they did this every five or ten minutes.

Others not so bad, would only stamp their feet. I did nothing for them. Dr. Bridge came on the 16th, examined the cattle and diagnosed the trouble to be in the clover-hay, which was at once stopped. I gave each cow a large dose of Mag. sulph., followed by tonics. On the 17th, I saw for the first time the cows come out of the stable; here was where the trouble showed itself the most, coming from the dark stable to the light, during the first few steps the fetlocks would hardly bear them, they would turn and almost throw them down. As soon as they came to the light they would start back, stare around, blink their eyes and were not able to distinguish objects; one cow especially could hardly get through an opening twelve feet high by fifteen wide, making several attempts before getting out, she would start out then step back, start again, bend down until belly almost touched the ground, after doing this several times she got down upon her knees, rested in that position a few seconds, then got up and passed out without any trouble and went to eating. She did this three mornings in succession, before improving the whole herd went through these actions more or less in getting out, all were very thirsty and drank a great deal of water.

Of the two which were in the stable the one that was the weakest, but able to stand, got down in the night and died while I was looking at the others; she ate some hay and drank water early in the morning. the other one still down but eating and milking some.

I saw them again in the evening they were apparently much better after standing out all day, the stiffness and trembling was improved. On the second day they came out of the stable much the same as the day before but did not seem so nervous and it soon passed off after being out. The cow that had been down since the 14th, died during the night. I held an autopsy on the two which had just died; the first thing noticed upon opening them was a peculiar smell, different from any I had ever encountered in any other cadaver, as near as I can describe it, it was like rotten eggs.

I found all the internal organs in a normal healthy condition except a very slight inflamation of the brain and lesions of the stomachs; the whole affection seemed localized in the stomachs. The mucous membrane was almost entirely separated from the wall of the rumen and manyplies leaving it of a purpleish hue, looking very much as if having lain in water for several days. Both cows were in the same condition.

On the 19th, I saw them come out of the stable, the whole herd came out better than yesterday. Having hay in bunches about the lot, the cows chased and hooked each other around in their eagerness to get at it. An old cattle dealer that was looking at them at the time remarked, "that those cows were not sick, as sick cows would not eat in that way. On the 20th, when the cows were coming out of the stable I detected one that was very stary, I had here at once put back and gave mag. sulp., one lb., the herd generally seemed more stiffened up, not so well as yesterday. Thinking the tonic too strong for the weak condition the stomach was in, I changed it. I put lime in the drinking water as they were drinking an immense quantity of it and then bloating up, they would go to the water-trough looking thin, in a half hour they would be bloated up so as to be hardly able to get around.

Some of the man's neighbors lold him if he would only turn his cows out upon good pasture that would be the end of it, he thought he would try one first, this one had been very bad but was improving nicely when he turned her into his lawn for about one hour, where the grass was good, she then went to the water and drank, but did not get ten feet from the trough until she fell; she lived for a week after but did not get upon her feet, at the end of that time seeing no improvement, I had her killed. On the 21st, I did not see them come out of the stable, they bloated some after drinking water; on the 22d, they were all doing better. On the 23d, one old cow which I had advised being kept out of the stable all the time had been getting along nicely, the night being cold the owner put her in, she got down during the night, could not rise in the morning and lived two days when he killed her.

I did not see them again until the 27th, they were then all doing well, picking up in flesh and giving almost as much milk as before. Of the four cows which died, as they were his best milkers, he was feeding them more than any the rest.

Dr. Hoskins was of the opinion that the trouble was due to some mould fungus or germs which had produced the result, similar, in many instances to the so-called cerebro-spinal meningitis in horses. Dr. Rayner was sure that it was due solely to the feeding of malt-dust, but could not explain what there could be in the food that would produce the results cited. Dr. Kooker gave the composition, and manner of preparation of what is sold as malt-dust. The cases cited were of great interest to the members present, and remarks were made and questions asked by everyone present. The opinions of the majority being that the trouble was the result of mould. The subject of Mycotic Stomatitis was brought up for discussion. There were over 300 cases reported as having been treated by members present. Dr. W. B. E. Miller stated that it was due to a vegetable irritant likely of the ergot groups. All cases were of a mild character which yielded readily to treatment. No fatal cases reported.

The treasurer reported collections for evening $39.00, against which orders for printing, rent, etc., were drawn to the amount of $38.25. The President, Dr. Hoskins, announced that a remedy would be discussed at the next meeting. Adjourned.

W. S. Kooker, *Secretary*.

